# Treatment of Tungiasis with Dimeticone: A Proof-of-Principle Study in Rural Kenya

**DOI:** 10.1371/journal.pntd.0003058

**Published:** 2014-07-31

**Authors:** Marlene Thielecke, Per Nordin, Nicholas Ngomi, Hermann Feldmeier

**Affiliations:** 1 Institute of Microbiology and Hygiene, Campus Benjamin Franklin, Charité University Medicine, Berlin, Germany; 2 Skaraborg Institute for Research and Development, Skövde, Sweden; 3 African Population and Health Research Center, Nairobi, Kenya; University of California San Diego School of Medicine, United States of America

## Abstract

Tungiasis (sand flea disease) is a neglected tropical disease, prevalent in resource-poor communities in South America and sub-Saharan Africa. It is caused by an inflammatory response against penetrated female sand fleas (*Tunga penetrans*) embedded in the skin of the host. Although associated with debilitating acute and chronic morbidity, there is no proven effective drug treatment. By consequence patients attempt to remove embedded sand fleas with non-sterile sharp instruments, such as safety pins, a procedure that represents a health threat by itself. In this proof-of-principle study we compared the topical application of a mixture of two dimeticones of low viscosity (NYDA) to the topical application of a 0.05% solution of KMnO4 in 47 school children in an endemic area in rural Kenya. The efficacy of the treatment was assessed during a follow up period of seven days using viability signs of the embedded parasites, alterations in the natural development of lesion morphology and the degree of local inflammation as outcome measures. Seven days after treatment, in the dimeticone group 78% (95% CI 67–86%) of the parasites had lost all signs of viability as compared to 39% (95% CI 28–52%) in the KMnO4 group (p<0.001). In the dimeticone group 90% (95% CI 80–95%) of the penetrated sand fleas showed an abnormal development already after 5 days, compared to 53% (95% CI 40–66%; p<0.001) in the KMnO4 group. Seven days after treatment, signs of local skin inflammation had significantly decreased in the dimeticone group (p<0.001). This study identified the topical application of dimeticones of low viscosity (NYDA) as an effective means to kill embedded sand fleas. In view of the efficacy and safety of the topical treatment with dimeticone, the mechanical extraction of embedded sand fleas using hazardous instruments is no longer warranted.

## Author Summary

Tungiasis (sand flea disease), a parasitic skin disease, causes important morbidity, and eventually leads to mutilation of the feet. Hitherto, the only effective treatment is the surgical extraction of embedded sand fleas. In the endemic areas this is done using inappropriate sharp instruments and causes more harm than good. We identified the three last abdominal segments of *Tunga penetrans* which protrude through the skin and through which the parasite breathes, defecates, and expels eggs - as an Achilles heel of embedded sand fleas. In a proof-of-principle study we investigated whether this Achilles heel is vulnerable to dimeticone with a low viscosity and a high creeping property. We randomized the left and the right feet to either receive a topical application of KMnO4 (the standard treatment in Kenya) or of dimeticone. The major outcome measure was the absence of viability signs of the treated sand fleas. The study shows that the topical application of a mixture of two dimeticones (NYDA) effectively kills embedded sand fleas within seven days. Since dimeticones are considered to be wholly non-toxic and are not expensive the new treatment could become a means to control tungiasis-associated morbidity on the population level.

## Introduction

Tungiasis (sand flea disease) is a neglected tropical disease frequent in South America, The Caribbean and in sub-Saharan Africa. [1], [2], [3]. It is prevalent in resource-poor rural and urban communities, where animal reservoirs are present and people live in poverty [2], [4], [5], [6], [7], [8]. In the last decade, tungiasis has re-emerged in East Africa in epidemic dimensions [9]. In 2010, Ahadi Kenya Trust, a non-governmental organization, reported several hundred thousand cases of tungiasis in Kenya alone, of which the majority were children [4], [10], [11].

Sand flea disease is the result of an intense inflammatory response against penetrated sand fleas embedded in the skin of the host. The mechanisms underlying the inflammation are complex and only partially understood [11], [12], [13]. Immediately after a successful penetration the female sand flea starts to hypertrophy reaching the size of a pea after 10 days [14]. Through its abdominal rear cone the parasite remains in contact with the environment [14]. The tiny opening in the skin (250 to 500 µm) is needed for copulation with male sand fleas, breathing, defecation and expelling eggs [14]. After expulsion of all eggs the female sand flea dies in situ and is discarded from the epidermis by tissue repair mechanisms [14].

Although by its nature a self-limiting infection, tungiasis is actually a debilitating disease in endemic areas [15]. Sequels are common and are related to repeated and severe infection. They include acute and chronic inflammation of toes, deformation and loss of toe nails, fissures and lymphoedema [11].

Bacterial super-infection is almost invariably present [13]. It increases the inflammation and leads to intense pain [16]. If embedded sand fleas are removed by using inappropriate sharp instruments, severe mutilation of the feet may develop including deep ulcers, gangrene and loss of toes [15]. Septicaemia has also been described [17] and tetanus is a known deadly sequel in non-vaccinated individuals [18].

Hitherto, the only effective treatment is the surgical extraction of embedded sand fleas under sterile conditions in medical facilities. However, in the endemic areas patients do not have access to appropriately equipped health centers and therefore use any kind of sharp instruments (safety pins, sewing needles, hair pins, sharpened pieces of wood, etc.) to remove embedded sand fleas. Attempts to remove the embedded parasites by using a sharp instrument, invariably causes a (micro) hemorrhage [9]. As the same instrument is frequently used to remove embedded sand fleas from different persons, this procedure increases the risk of the transmission of blood-borne pathogens, such as hepatitis B and C virus [19].

In an act of desperation, patients may apply toxic substances to the skin with the intention of killing the embedded parasites. In Brazil and Madagascar, for instance, kerosene, used petrol, and insecticides are used [9], [20]. In rural Uganda, a crop pesticide used in tomato cultivation is applied (H. Feldmeier, unpublished observation 2013).

In the absence of safe and effective treatment options, Ahadi Kenya Trust recommends to bath the feet in a 0.05% solution of potassium permanganate (KMnO4) for 10 minutes [10]. However, the efficacy of this approach is not known. In Brazil several antihelminthic compounds, including ivermectin, have been tested, but none proved to be a really effective [21].

Dimeticones are silicone oils of low viscosity with a low surface tension and excellent creeping properties. They are highly effective against head lice [22]. The substance creeps into the tracheae of head lice and leads to lethal asphyxia within one minute [23]. The mode of action is purely physical. Dimeticones are biochemically inert and are not absorbed when applied to the skin or swallowed [24]. They are neither carcinogenic nor teratogenic and are considered wholly non-toxic [24].

Previous observation in rats infested with *T. penetrans* showed that if a drop of a solution of two dimeticones of low viscosity (NYDA) was applied on top of the protruding rear cone of an embedded sand flea, the parasite rapidly lost signs of viability (H. Feldmeier, unpublished observation 2011). Based on this observation we decided to investigate the efficacy of the dimeticone for the treatment of tungiasis in a proof-of-principle study in rural Kenya. The results show that wetting the skin of the feet with dimeticones with low viscosity effectively kills embedded sand fleas and reduces tungiasis-associated inflammation within seven days.

## Materials and Methods

### Study area and study population

The study was performed in Gatundu North District, central Kenya, approximately one hour north of Nairobi. Tungiasis is endemic in this region. People live in small hamlets in houses made of wood or bricks. Families earn their living from subsistence farming. Most households possess animals, dogs, chicken and pigs. The animals live on the compound or are brought back to it in the night. Living conditions are generally very poor.

The study participants were school children aged five to sixteen years enrolled at the public Kiamwangi Primary School and Ikuma Primary School, which are situated five km to each other. The classrooms consist of simple houses without a solid floor. Both schools have a limited access to water, so that the schoolyards and rooms cannot be cleaned regularly. Most pupils wore worn-out sandals or walked barefoot. The study was carried out between January 10 and February 17, 2012. This period coincides with the high transmission period of *T. penetrans.*

### Study design

To allow comparison between the new approach (the application of the dimeticone) and the local reference procedure (bathing feet in a 0.05% solution of KMnO4), one foot was bathed in the KMnO4 solution for 10 minutes and to the other foot the dimeticone was applied three times during this period (see below). Since bathing a foot in a 0.05% KMnO4 solution changes the color of the skin into dark purple, neither the patient nor the examiner were blinded with regard to the treatment applied.

Individuals, aged ≥5 years, with at least one lesion in stage IIa – IIIa of the Fortaleza classification on each foot were eligible [14]. In IIa the sand flea is already completely embedded in the skin of the host and has started to hypertrophy [14]. Lesions in stage IIIa correspond to a fully developed parasite with a characteristic watchglass-like appearance. In this stage the female sand flea starts to expel eggs [14]. In stage IIIb egg expulsion stops, thereafter the sand flea dies and the lesion changes into stage IV: the lesion becomes crusted, viability signs become rare and eventually completely disappear [14]. Hence, sand fleas in stage IIa – IIIa are most suitable to assess viability and alterations in the normal development of the parasites.

The inclusion criterion for an eligible lesion was the presence of at least 2 out of 4 viability signs at the baseline examination: expulsion of eggs, excretion of a faecal thread, excretion of faecal liquid or pulsations/contractions of the parasite. Viability signs were determined using a handheld digital video microscope (eScope iTEZ, Hongkong, China) (see supplementary electronic material 1).

When several eligible lesions were present on one foot only those (at most three) were selected for evaluation that allowed a clear discernment of the developmental stage of the embedded parasite and a quantification of the inflammatory response around the lesion. Hence, lesions occurring in cluster and lesions which the patient had attempted to manipulate were excluded. Other exclusion criteria were: Presence of gross inflammation, abscess or ascending lymphangitis or lymphedema on either foot. Children with such complications of tungiasis were referred to the nearest health facility for treatment.

For practical reasons we decided to treat always the same foot with dimeticone and KMnO4, respectively. At the beginning of the study a coin was tossed for randomizing the two treatments. This resulted in application of the dimeticone to the left foot and of KMnO4 to the right foot. Children were informed not to manipulate the lesions during the next seven days.

Before each examination the feet of the participants were washed properly with water and soap and dried with a clean towel. Then, the left foot was wetted with NYDA up to the ankle three times within 10 minutes. In the interval, the foot was kept in an upright position to allow surplus dimeticone to evaporate. Simultaneously, the right foot was put into a bucket containing a 0.05% KMnO4 solution, and remained there for 10 minutes. After sun drying the right foot, vaseline was applied to compensate the desiccation of the skin caused by KMnO4. The immersion of the foot in 0.05% KMnO4 for 10 minutes and the subsequent oiling with vaseline is the standard procedure applied by Ahadi Kenya Trust. After treatment the children were allowed to continue their daily activities.

The lesions were monitored daily for viability signs and the abnormal development of the embedded parasite for a total of seven days. One week reflects the period of normal development of a sand flea from stage IIa to stage IIIa [14]. Thereafter, it looses its characteristic watchglass-like appearance, but does not increase in size anymore [14]. Hence, abnormalities in development are difficult to be detected.

In order to detect a change of tungiasis-associated inflammation an inflammation score was developed. In addition to the classic signs of local inflammation (erythema, oedema and warmness) the score included the presence of suppuration, ulcers and fissures as well as itching and pain. The inflammation score ranged from 0 to 27 points [25].

**Figure 1:**
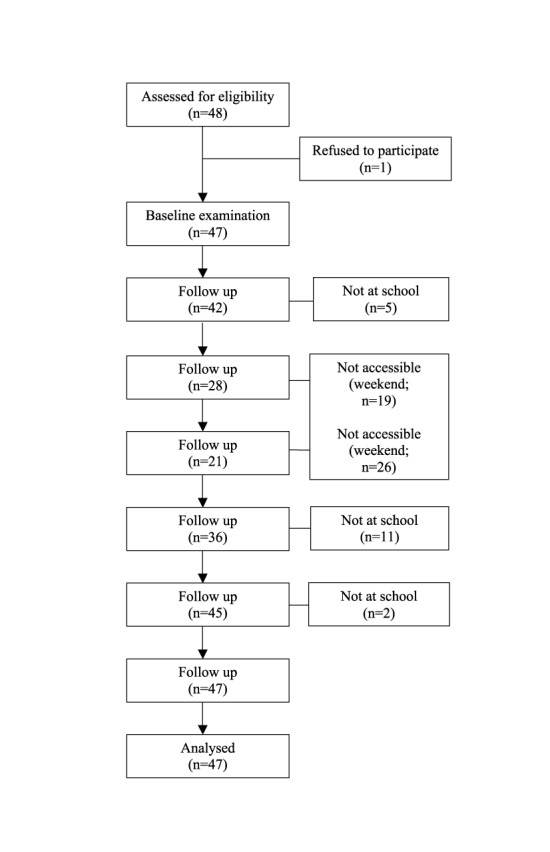
Flow diagram.

In total, 48 participants were recruited and 47 were randomized. The flow diagram is shown in Figure 1.

### Outcome measures

Two major outcome measures were defined. First, the proportion of viable embedded sand fleas which lost viability signs after seven days of follow-up. An embedded sand flea was considered to be dead when none of the four viability signs (expulsion of egg, excretion of faecal thread, excretion of faecal liquid, pulsations/contractions) was detected during 15 minutes of observation with the digital handhold video microscope on two consecutive follow-up examinations. Videos were recorded and reviewed in the evening of the examination day (see supplementary electronic material 1). Second, the proportion of embedded sand fleas in which the normal development was interrupted. We defined a development as abnormal, when the lesion did not change its size within two consecutive follow ups and/or morphological abnormalities developed, e.g. discoloring or desiccation of the abdominal rear cone [14].

A secondary outcome measure was the intensity of local inflammation, as assessed semi-quantitatively by the inflammation score. The observation units for all outcome measures were single sand flea lesions.

### Statistical analysis

The sample size calculation was based on the following assumptions: with a level of confidence set at 95% together with a power of 90% assuming equal number of lesions in treatment and control group, 45 lesions in each group were needed to determine a difference of 35% in the major outcome measure between the two treatments assuming a 40% effect of the standard treatment.

Fisher's exact test was used to compare proportions. General estimation equations were used to analyze the evolution of the inflammation score during the observation period.

### Ethical considerations

The study was approved by the Ethics Committee of the Ministry of Health, Nairobi (MMS/ADM/3/8/Vol 111), and was registered at Controlled-trials.com (ISRCTN: 91405042). The study was performed in accordance with the ethical standards of the Ethics Committee of the Ministry of Health, and with the Declaration of Helsinki as amended 2013 by the World Medical Association. Informed written consent was obtained from the guardians of the participants in English before starting the study. For ethical reasons no controls were included. During the study, food was provided free of charge to the participants. At the end of the study, any remaining viable sand fleas were removed under sterile conditions and the wounds were dressed following standard procedures. All patients received a new pair of closed solid shoes.

## Results

### Baseline characteristics

The baseline characteristics of the feet of the 47 participants are summarized in Table 1. None of the variables differed significantly between the two feet. In the NYDA group, 88 lesions were included in the study, in the KMnO4 group 82.

**Table 1:** Demographic and clinical data of study participants at baseline.

	Treatment applied	
Variable	NYDA (left foot)	KMnO4 (right foot)
Median number of lesions on respective foot (range)^a^	25 (8–112)	25 (6–107)
Median of viable lesions (range)^b^	3 (1–29)	2 (1–25)
Median of non-viable lesions (range)^c^	3 (0–30)	2 (0–36)
Median of manipulated lesions (range)^d^	18 (6–53)	18 (4–54)
Number of viable lesions included in the study^e^:	88	82
stage IIa	52	51
stage IIb	35	31
stage IIIa	1	0

a total number of viable, non-viable and manipulated sand flea lesions.

b sand flea lesions in stage I to IIIb, according to the Fortaleza Classification.

c lesions in stage IV and V, according to the Fortaleza Classification.

d lesions manipulated with a sharp instrument by the patient himself or a caregiver.

e maximum of 3 lesions per foot (see material and methods).

### Major outcome measures

Table 2 shows the efficacy of treatment based on the disappearance of viability signs. Already three days after application of dimeticone 50% of the parasites lost all viability signs (efficacy = 50%), whereas the efficacy in the KMnO4 group was 14% (p<0.001). At day 7 the efficacy was 78% (95% CI 67–86%) after treatment with dimeticone and 39% (95% CI 28–52%) after treatment with KMnO4 (p<0.001); a difference of 39% (95% CI 23–54%). In the dimeticone group, lesions in an early stage of development lost viability signs more often than lesions in later stages (efficacy = 88% (95% CI 75–95%) versus 65% (95% CI 47–79%) at day 7 (p = 0.01)). In the KMnO4 group, there was no difference between lesions in early and later stages of development.

**Table 2:** Efficacy of treatment based on viability of embedded sand fleas.

			Treatment			
		NYDA viable/total lesions (%)	Efficacy (%)^a^	KMn04 viable/total lesions (%)	Efficacy (%)^a^	p-value^b^
Baseline	All lesions^c^	89/89 (100%)	0%	82/82 (100%)	0%	
	- early stages (IIa)^d^	52/52 (100%)	0%	52/52 (100%)	0%	
	- later stages (IIb–IIIa)^d^	37/37 (100%)	0%	30/30 (100%)	0%	
Day 3	All lesions^c^	27/54 (50%)	50%	43/50 (86%)	14%	<0.001
	- early stages (IIa)^d^	12/28 (43%)	67%	29/33 (88%)	12%	<0.001
	- later stages (IIb–IIIa)^d^	15/26 (58%)	42%	14/17 (82%)	18%	0.10
Day 5	All lesions^c^	33/72 (46%)	54%	43/58 (74%)	26%	0.001
	- early stages (IIa)^d^	18/43 (42%)	58%	26/37 (70%)	30%	0.01
	- later stages (IIb–IIIa)^d^	15/29 (52%)	46%	17/21 (81%)	19%	0.04
Day 7	All lesions^c^	19/86 (22%)	78%	43/71 (61%)	39%	<0.001
	- early stages (IIa)^d^	6/49 (12%)	88%	27/45 (60%)	40%	<0.001
	- later stages (IIb–IIIa)^d^	13/37 (35%)	65%	16/26 (62%)	38%	0.04

a proportion of parasites which lost all viability signs.

b dimeticone versus KMnO4 treatment.

c The total number of lesions examined varied at follow up examinations, because some participants could not be examined at the days foreseen, especially at the weekends (see flow diagram).

d according to the Fortaleza classification.

The effect of treatment on the morphological development of the lesions is shown in Table 3. Already after 5 days in the dimeticone group 90% (95% CI 80–95%) of sand flea lesions showed an abnormal development as compared to 53% (95% CI 40–66%) (p<0.001) in the KMnO4 group.

**Table 3:** Efficacy of treatment based on the morphological development of sand flea lesions.

		Treatment applied		
		NYDA	KMnO4	p-value^b^
		Abnormal development/total lesions (%)^a^		
Day 3	All lesions^c^	41/54 (76%)	22/50 (44%)	<0.001
	- early stages (IIa)^d^	19/28 (68%)	12/33 (36%)	0.021
	- later stages (IIb–IIIa)^d^	22/26 (85%)	10/17 (59%)	0.080
Day 5	All lesions^c^	65/72 (90%)	31/58 (53%)	<0.001
	- early stages (IIa)^d^	40/43 (93%)	20/37 (54%)	<0.001
	- later stages (IIb–IIIa)^d^	25/29 (86%)	11/21 (52%)	0.012
Day 7	All lesions^c^	79/86 (92%)	45/71 (63%)	<0.001
	- early stages (IIa)^d^	45/49 (92%)	27/45 (60%)	<0.001
	- later stages (IIb–IIIa)^d^	34/37 (92%)	18/26 (69%)	0.040

a see definition in materials and methods.

b dimeticone versus KMnO4 treatment.

c The total number of lesions examined varied at follow up examinations, because some participants could not be examined at the days foreseen, especially at the weekends (see flow diagram).

d according to the Fortaleza classification.

**Figure 2:**
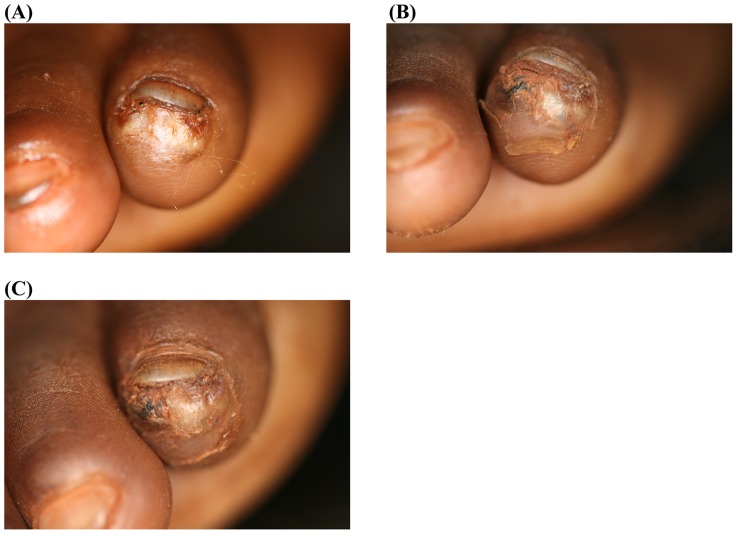
Photo series of two lesions located next to the nail rim of the fifth toe; treatment with dimeticone. (A) Baseline: Two sand flea lesions in stage IIIa are located next to each other with the characteristic watchglass-like elevation. The abdominal cone is the circular brownish protrusion in the center of the lesions. (B) Day 3: The abdominal cones have changed in a brownish-black crust, the watchglass-like elevations have vanished and the lesions have dried out. Desquamation of the stratum corneum around the lesions has started. No signs of viability were detected. (C) Day 7: The appearance of the lesions has not changed; desquamation has slightly increased.

**Figure 3:**
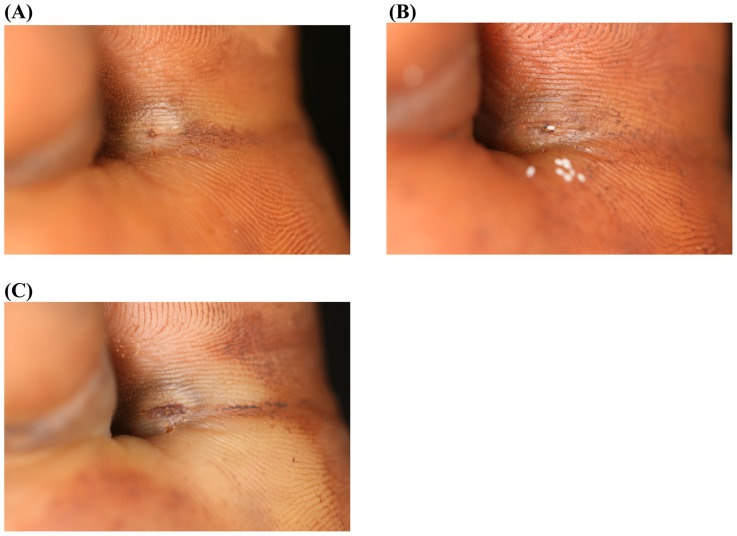
Photo series of a lesion located at the base of the first toe; treatment with KMnO4. (A) Baseline: A lesion in stage IIIa with a diameter of 10 mm at the base of the first toe. The abdominal cone is the circular brownish protrusion in the center of the elevation. The dermal papillae next to the lesion contain faecal material expelled by the parasite. (B) Day 3: The sand flea has expulsed several eggs (white oval dots). One of the eggs is in progress of being expelled. The appearance of the lesion has not changed. (C) Day 7: The lesion has retained its size and remains elevated. Recently excreted faecal material has spread into the dermal papillae next to the lesion, another indicator that the parasite remained viable.

**Figure 4:**
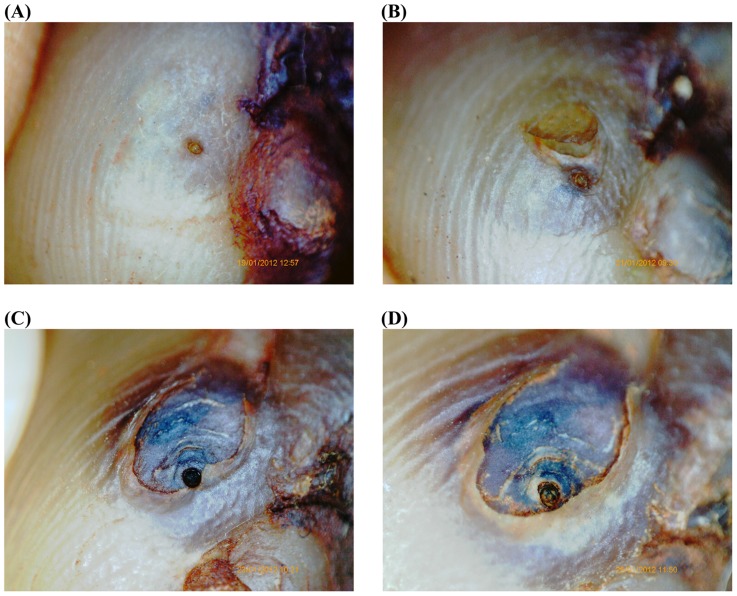
Photo series of a lesion documented by the digital handhold video microscope at 200 fold magnification; treatment with dimeticone. (A) Baseline: Lesion in stage IIb. The abdominal cone is the circular brownish protrusion in the center. The cone is surrounded by a slightly elevated circle. The dark area on the right is part of the toe nail. (B) Day 3: The abdominal cone has changed in a brownish crust. The stratum corneum covering the embedded parasite has started to desquamate. No viability signs detectable. (C) Day 5: The rear cone has changed into a black crust. The desquamation has significantly enlarged. The uncovered intersegmental skin of the abdomen of the parasite has turned into dark-purple. (D) Day 7: The appearance of the lesion has remained similar; desiccation and desquamation have continued.

**Figure 5:**
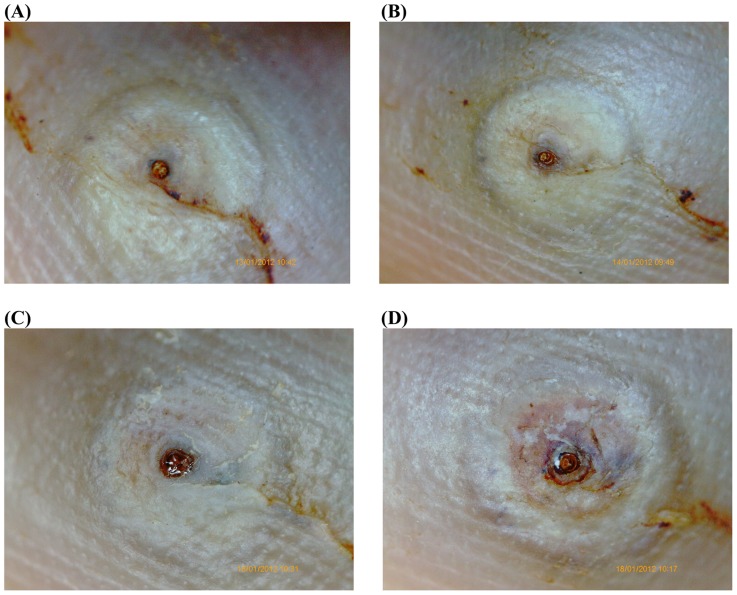
Photo series of a lesion documented by the digital handhold video microscope at 200 fold magnification; treatment with KMnO4. (A) Baseline: Lesion in stage IIIa. The abdominal cone is the circular brownish protrusion surrounded by the characteristic watchglass-like elevation. The curved line is faecal material of the parasite that has spread into dermal papillae. (B) Day 3: The embedded parasite has grown slightly and the convex elevation is more embossed. The abdominal cone is still brownish and shining. (C) Day 5: The appearance of the lesion has not changed. Faecal liquid is excreted through the abdominal cone and appears as a clear, light-reflecting “pond” on the top of the cone. (D) Day 7: The abdominal cone is still brownish and shining. The lesion has a convex double-rim appearance. Two viability signs (pulsation of the parasite and excretion of liquid) were present at this moment.

Figure 2A–C and 3A–C show the macroscopic development of lesions after the treatment with dimeticone or KMnO4, respectively. Figure 4A–D and 5A–D depict the microscopic development of lesions after treatment as seen through the digital handhold video microscope.

### Inflammation score

In the dimeticone group the inflammation score decreased from a median of 6.0 at baseline to a median of 4.75 at day 7. In contrast, in the KMnO4 group, the inflammation score increased (median 4.5 versus 5.0). Both differences were significant (p<0.0001 and p = 0.009, respectively).

### Ancillary findings

During the study period three sand fleas were extracted by the participants or their caregiver in the NYDA group and 11 in the KMnO4 group.

## Discussion

Tungiasis, a wide spread neglected tropical disease, is prevalent in resource-poor rural and urban communities, where animal reservoirs are present and people live in poverty [2], [4], [5], [6], [7], [8]. Elimination of sand flea disease is not possible as long as the precarious living conditions, which are characteristic of the endemic areas, prevail and animal reservoirs exist.

Taking into consideration the high prevalence of tungiasis, the absence of appropriate infrastructure in the endemic areas and the health hazards associated with the traditional treatment, there is an urgent need for a safe and effective drug treatment. Recently, dimeticones have emerged as highly effective chemicals against ectoparasites such as head lice [26]. Since dimeticones have a purely physical mode of action and are considered to be non-toxic, they have become the standard treatment of pediculosis capitis in Europe [22].

We considered the last abdominal segments of an embedded sand flea, which protrude through the skin by forming a miniature cone and through which the parasite breathes, defecates and excretes eggs, as an Achilles heel, which can be targeted by dimeticone. Since the opening leading to internal organs measures less than 1 mm, we decided to use a combination of two dimeticones of very low viscosity with a low surface tension and excellent creeping properties (NYDA) [23].

We defined a set of viability signs of embedded sand fleas detectable through a handhold digital video microscope. We used the presence of viability signs as the major outcome measure and compared the efficacy of a 0.05% solution of KMnO4 – the standard treatment used in mass campaigns in Kenya – to wetting the foot with dimeticone three times during a period of 10 minutes. The observation period was limited to seven days, since a certain number of embedded sand fleas will die even without any intervention during this period [14].

After 7 days, 78% of the lesions did not show any sign of viability in the dimeticone group, whereas the proportion was 39% in the KMnO4 group. True efficacy of a 0.05% solution of KMnO4 alone may be lower since KMnO4 is a disinfectant and has no insecticidal properties. It is unlikely that KMnO4 diluted in water will creep into vital organs of embedded sand fleas through the parasite's abdominal cone. Presumably, the observed effect in the KMnO4 treated lesions was due to the vaseline which was applied to the skin for cosmetic reasons (because bathing the feet in KMnO4 makes the skin rough and cracked). Applied on the skin, vaseline rapidly turns into oil, particularly in hot climate countries. Liquid fatty acids of the vaseline may thereby creep into the abdominal rear cone and suffocate the parasite.

Interestingly, the efficacy of dimeticone to kill embedded sand fleas depended on the stage of development: parasites being in an early stage of development were more susceptible than those who had already fully developed (efficacy = 88% versus 66%). This is plausible, since embedded sand fleas increase their size by a factor of approximately 2000 within 6–7 days during the development from stage IIa to stage IIIa [14]. Such a rapid growth requires an intense metabolism, which in turn needs constant supply of oxygen. During the early stages of development supply of oxygen might be at a critical limit. This makes the parasite vulnerable for suffocating compounds such as low-viscosity.

Since it is important to kill sand fleas as soon as they have penetrated in order to prevent the development of clinical pathology [16], the enhanced effect of dimeticone on early developmental stages is an additional advantage. The early death of the embedded parasite will also prevent the expulsion of eggs – which starts about one week after penetration – and, thereby, may have an impact on transmission.

92% of the embedded fleas treated with dimeticone showed an abnormal development. This could indicate that no (or fewer) eggs are produced and released into the environment. Hence, if applied on the population level, treatment with dimeticones could have even an impact on the off-host cycle of the parasite, possibly resulting in lower attack rates over time.

In the dimeticone group, the inflammation score started to decrease after 3 days and became significantly lower after 7 days, whereas in the KMnO4 group the inflammation slightly increased. It is conceivable that the resolution of inflammation reflects the rapid death of the parasites. Previous studies have shown that tungiasis-associated inflammation comes to a halt and tissue repair mechanism begins, when the parasites are dead [25], [27].

**Figure 6:**
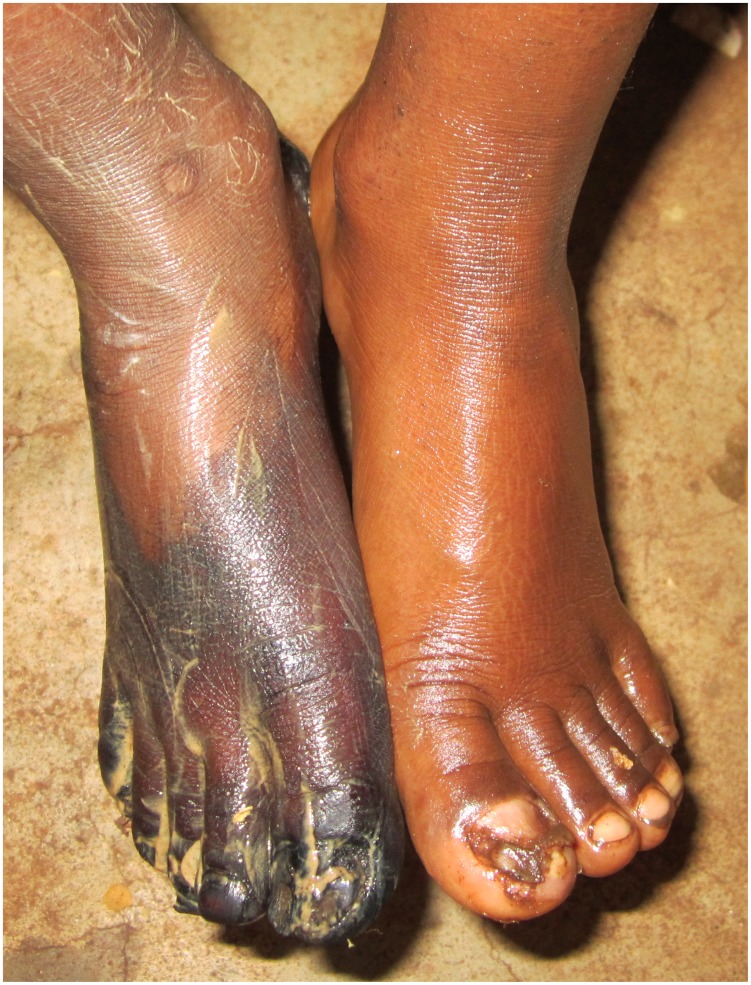
Left and right foot after the application of the dimeticone and KMnO4, respectively. The dark coloring of the right foot is due to KMnO4. The yellow jelly on the right foot is vaseline being in the process of dissolution.

Another indicator of the efficacy of the dimeticone was that in the course of the study 11 sand fleas were extracted from the feet treated with KMnO4 by the patients themselves, whereas in the NYDA treated feet only 3 sand fleas were removed. Similarly, when the study participants were asked at the end of the study about their satisfaction, only 10 participants preferred KMnO4, but 37 preferred the dimeticone. Children also disliked that KMnO4 colored the skin into deep purple for a few days which led to teasing in school (Figure 6).

This study on the treatment of a neglected parasitic disease is particularly in the sense that an Achilles heel of the parasite was identified first and then a compound was identified that is able to target the vulnerable body part. The abdominal cone which protrudes through the skin and through which the parasite breathes, defecates, excretes liquids and expels eggs was considered to be an ideal target for a dimeticone with a low viscosity and excellent creeping properties.

Although this was a proof-of-principle study with a small number of units of observations, it can be concluded that the topical application of a mixture of two dimeticones (NYDA) comprises a promising approach to treat sand flea disease. The treatment can be performed by the patient himself with minimal input from the health sector. Hence, surgical extraction with all its associated complications is no longer warrantable. After the sand flea has died in situ, the inflammation resolved. Importantly, future resistance of the parasites against dimeticone treatment is highly unlikely to evolve, since the drug acts only physically.

## Supporting Information

Video S1 Embedded sand flea produces faecal thread. Video of an embedded sand flea in stage IIb using a handheld digital video microscope. The lightly brownish abdominal rear cone is magnified 200 fold. The cone is contracting and producing a black faecal thread. In the surrounding of the cone pulsations of the intestines are visible.
(MP4)
